# Hypotension prediction index guided goal-directed therapy to reduce postoperative acute kidney injury during major abdominal surgery: study protocol for a multicenter randomized controlled clinical trial

**DOI:** 10.1186/s13063-024-08113-w

**Published:** 2024-04-29

**Authors:** Javier Ripollés-Melchor, Laura Carrasco-Sánchez, José L. Tomé-Roca, César Aldecoa, Andres Zorrilla-Vaca, Juan V. Lorente-Olazábal, María J. Colomina, Ana Pérez, Juan I. Jiménez-López, Rosalía Navarro-Pérez, Alfredo Abad-Gurumeta, Manuel I. Monge-García, the HYT Study Group, Alicia Ruiz-Escobar, Paula Fernánez-Valdes-Bango, Ángel V. Espinosa, María A. Fuentes Pradera, Azahara Cortes Rueda, Ana B. Adell-Perez, Iker Agarrista-Aguirrezabala, Marta Amelburu-Egoscozabal, Josu Ariño-Larrañaga, Aitor de Haro-Ferrari, Manuel Eced-Sanchez, Maria Eizaguirre-Cotado, Alazne Enparantza-Aiestaran, Cristina Garcia-Fernandez, Carmen Garicano-Goldaraz, Nuria Gonzalez-Jorrin, Silvia Gonzalez-Santos, Andrea Lara-Jimenez, Edurne Lodoso-Ochoa, Amaia Lopetegui-Aizpurua, Jorge Mendoza-Sorrondegi, Antia Osorio-Lopez, Amaia Uria-Azpiazu, Virginia Serrano Zarcero, Ane Abad-Motos, Hussein Abu Khudair, Rocío Venturoli Ojeda, Alvaro Mingote-Lladó, Diego Gutiérrez-Martínez, Alberto Gutiérrez-Martínez, Ana Moreno Martín, Javier García-Fernández, Bartolomé Fernández Torres, Ignacio Martin de Pablos, Javier María Valiente Lourtau, Estefanía Peralta Espinosa, Jose Manuel Prieto Gutiérrez, Víctor Lama Paniego, Ángel Cárdenas Duque, Ángel Becerra-Bolaños, Carmen A. Focaccio-Tejada, Aurelio Rodríguez-Pérez, Sergio López-Ruiz, Lucía Valencia-Sola, Patricia Galán-Menéndez, Daniel García-López, Marc Sadurni-Sarda, Hugo Rivera-Ramos, Irene Romero-Bhathal, Laura Castelltort-Masco, Carlos E. Moreno-Martinez, Jesus Carazo-Cordobes, Leire Larrañaga-Altuna, Eva Bassas-Parga, Ana Tejedor-Navarro, Jesús Fernanz-Antón, Marta Garcia-Martínez, Aleix Carmona-Blesa, Elisenda Pujol-Rosa, J. Miquel Moncho-Rodríguez, Luisa F. Cueva-Castro, Astrid M. Batalla-González, Marta Giné-Servén, María M. Bastitta, Laura A. Pardo-Pinzón, Alex Arjona-Navarro, Gonzalo Azparren-Cabezón, Alejandro Gutiérrez-Marqués, Alejandro García-Rodriguez, Adriano Paz-Martín, Daniel García-López, Gina Lladó-Jordan, José L. Rábago-Moriyón, Ceferina Suárez-Castaño, Ana Bolado-Álvarez de Eulate, Gabriel Escudero-Gómez, Julia Castaño-Álvarez, Eduardo Larraz-Mármol, Rodrigo Sancho-Carrancho, Marta Alonso-Fernández, Irma M. Barrio-Pérez, Nel González-Argüelles, Sara Martínez-Álvarez, Adriana I. Reyes-Echeverría, Guillermo Tejón-Pérez, Ángela Pascual-Casado, Pablo Garmilla-Ezquerra, Angel Villar Pellit de la Vega, Santiago Seco Gordillo, Ana M. Quintero Moreno, Peña Gómez Domínguez, Irene Mojarro, Jordi Llorca García, Francisco J. Cañas Perea, Cristina Prat Llimargas, Pere Esquius Jofré, Santiago Montesinos-Fadrique, Gabriel Yanes, Alvaro Ocón-Moreno, Samuel Moreno-Jiménez, Alberto Quevedo-Gutiérrez, Miguel Medina-Martos, Laura Herrera-Lozano, Sandra L. Martín-Infantes, Carlos M. Palacios-Vega, Cesar P. Garcia-Bertini, Ángela Salinas-Moya, Rocío Venturoli, Ana Moreno, Adrián Muñoz, Pablo Lobato, Montserrat Mallol, Andrea Gutierrez, Antonio Guillen, Ana Mugarra, Berta Monleón, Ana Gimeno, Esther Romero, Eduardo Passariello, Carmen Beltran, Eva Rivas, Marta Ubré, Beatriz Tena, Óscar Comino, Iago Dieguez, Miren A. Echevarria-Correas, Maite Chasco-Ganuza, Mercedes Olvera-García, Alejando Arrabal, Marta Díaz, Marta Caballero-Milan, Guillermo Alonso-Nadal, Guillermo Puig-Sanz, Antonio-Jose Navarro-Garcia, Marc Cebria-Fondevila, Míriam Millan-Ruiz, Wanyi Li, Neus Sagartal, Tatiana Dam, Jaume Puig, L. Alós-Zaragozá, MA. Pallárdó-López, Marta Rosselló-Chornet, L. Munoz-Devesa, MJ. Hernández-Cádiz, J. Hernández-Laforet, R. Sanchis, Martin, E. Biosca-Pérez, Y. Fernández, Laura Vaquero, David Laguna, Alba Diaz, Alicia Bordell, Esther Aguado, Delia Velasco, Eugenio Ruiz, María Garcia-Matesanz, Irene Arranz, María Jesus Sanz de Leon, Maria-Jose Blanco, Gerardo Arias-Cuesta, Amal Azzam-López, María C. Martín-González, Isabel Ruíz-Torres, Pablo Racionero-González, Jimena Escobar-Tapias, Alba Gonzalo-Millán, Yolanda Diez Remesal, José L. Garrido, Anna Arnau-Bartés, Francesca Reguant-Corominas, Laura Dos-Santos-Carregal, Sabela del-Río, Gema Curado-Zafra, Paola Saiz-Sánchez, Isabel Paniagua-Pacheco, Ángela Morales-Cubero, José L. Garrido-Calmaestra, Miguel A. Valbuena-Bueno, Ana Pedregosa-Sanz, Santiago Abreu-Paradell, Pau Vallhonrat-Alcántara, Anna Alonso-Manzano, Carolina Palma, Martí Esteban-Fernández, Luis Nassar-Clavijo, Ahmad Alraqqab, Zaid Ayesh, Hussein Abu Khudair, Abderrahman Barhoum, Murad Al-Kharabsheh

**Affiliations:** 1 Infanta Leonor University Hospital, Madrid, Spain; 2 Fluid Therapy and Hemodynamic Monitoring Group of the Spanish Society of Anesthesiology and Critical Care (SEDAR), Madrid, Spain; 3 Universidad Complutense de Madrid, Madrid, Spain; 4 Althaia Xarxa Assistencial Universitària de Manresa, Manresa, Spain; 5 Doctoral Program in Medicine and Biomedical Sciences, University of Vic-Central University of Catalonia (UVic-UCC), Vic, Spain; 6 Institut de Recerca I Innovació en Ciències de La Vida I de La Salut a La Catalunya Central (IRIS-CC), Vic, Spain; 7 Virgen de las Nieves University Hospital, Granada, Spain; 8 Río Hortega University Hospital, Valladolid, Spain; 9 Brigham and Women’s Hospital, Boston, USA; 10 Juan Ramón Jiménez University Hospital, Huelva, Spain; 11 Bellvitge University Hospital, Barcelona, Spain; 12 Elche University Hospital, Elche, Spain; 13 Virgen del Rocío University Hospital, Seville, Spain; 14 Clinico San Carlos University Hospital, Madrid, Spain; 15 Jerez de La Frontera University Hospital, Jerez de la Frontera, Spain

**Keywords:** Hemodynamic ptimization, Major lective bdominal urgery, Early oal-irected emodynamic therapy, Fluid herapy, Hemodynamic onitoring, Postoperative omplications, Acute Kidney Injury, Mortality

## Abstract

**Background:**

Acute kidney injury (AKI) is a significant postoperative complication associated with increased mortality and hospital costs. Hemodynamic strategies, such as goal-directed therapy, might reduce AKI risk. Predicting and proactively managing intraoperative hypotension may be helpful. This trial aims to investigate if a preemptive hemodynamic strategy guided by the hypotension prediction index (HPI) can decrease the incidence of moderate-to-severe AKI within 30 days following major elective abdominal surgery.

**Methods:**

This is an open-label, controlled, multicenter, randomized clinical trial that involves daily patient follow-up until hospital discharge. Inclusion criteria are patients aged over 65 and/or categorized as ASA III or IV physical status, undergoing major elective abdominal surgery (general, urological, or gynecological procedures) via laparoscopic or open approach under general or combined anesthesia.

**Intervention:**

In the intervention group, hemodynamic management will be based on the HPI and the advanced functional hemodynamic variables provided by the Hemosphere platform and the AcumenIQ® sensor (Edwards Lifesciences). The primary outcome is the incidence of moderate-to-severe AKI within 7 days post-surgery. Secondary outcomes include postoperative complications and 30-day mortality.

**Discussion:**

This study explores the potential of HPI-guided hemodynamic management in reducing AKI after major elective abdominal surgery, with implications for postoperative outcomes and patient care.

**Trial registration:**

ClinicalTrials.gov NCT05569265. Registered on October 6, 2022.

## Administrative information

Note: the numbers in curly brackets in this protocol refer to the SPIRIT checklist item numbers. The order of the items has been modified to group similar items (see http://www.equator-network.org/reporting-guidelines/spirit-2013-statement-defining-standard-protocol-items-for-clinical-trials/).

**Table:** 

Title {1}	HYPOTENSION PREDICTION INDEX GUIDED GOAL DIRECTED THERAPY TO REDUCE POSTOPERATIVE ACUTE KIDNEY INJURY DURING MAJOR ABDOMINAL SURGERY: STUDY PROTOCOL FOR A MULTICENTER RANDOMIZED CONTROLLED CLINICAL TRIAL
Trial registration {2a and 2b}.	The trial has been registered on ClinicalTrials.gov under the identifier NCT05569265
Protocol version {3}	Version 1, April 2022
Funding {4}	No funding
Author details {5a}	Javier Ripollés-Melchor ^1–3^, Laura Carrasco-Sánchez ^2,4–6^, José L. Tomé-Roca^2,7^, César Aldecoa^2,8^, Andres Zorrilla Vaca^2,9^, Juan V. Lorente-Olazábal^2,10^, María J. Colomina^2,11^, Ana Pérez^2,12^, Juan I. Jiménez-López^2,13^, Rosalía Navarro-Pérez^2,14^,Alfredo Abad-Gurumeta^1,3^ Manuel I. Monge-García ^2,15^ on behalf of the HYT Study Group Infanta Leonor University Hospital, Madrid, Spain 2.Fluid Therapy and Hemodynamic Monitoring Group of the Spanish Society of Anesthesiology and Critical Care (SEDAR) 3.Universidad Complutense de Madrid, Madrid, Spain 4.Althaia Xarxa Assistencial Universitària de Manresa, Manresa, Spain 5.Doctoral Program in Medicine and Biomedical Sciences, University of Vic-Central University of Catalonia (UVic-UCC), Vic, Spain 6.Institut de Recerca i Innovació en Ciències de la Vida i de la Salut a la Catalunya Central (IRIS-CC), Vic, Spain 7.Virgen de las Nieves University Hospital, Granada, Spain 8- Río Hortega University Hospital, Valladolid, Spain 9.Brigham and Women's Hospital, Boston, USA 10.Juan Ramón Jiménez University Hospital, Huelva, Spain 11.Bellvitge University Hospital, Barcelona, Spain 12.Elche University Hospital, Elche, Spain 13.Virgen del Rocío University Hospital, Seville, Spain 14.Clinico San Carlos University Hospital, Madrid, Spain 15.Jerez de la Frontera University Hospital, Jérez de la Frontera, Spain
Name and contact information for the trial sponsor {5b}	**Javier Ripollés-Melchor** **Ripo542@gmail.com** **SEDAR** **Sociedad Española de Anestesiología, Reanimación y Terapéutica del Dolor** **C/ José Abascal n° 46, 1°A, 28003 Madrid** **Tel.: 914 419 099** **Email: secretaria@sedar.es**
Role of sponsor {5c}	**The sponsor (SEDAR) played no part in study design; collection, management, analysis, and interpretation of data; writing of the report; and the decision to submit the report for publication**

## Introduction

### Background and rationale {6a}

Intraoperative hypotension (IOH) has been consistently associated with postoperative complications, such as acute kidney injury (AKI) [1]. Therefore, preventing IOH has the potential to reduce postoperative organ dysfunction, highlighting the importance of early intervention [2]. The hypotension prediction index (HPI) is an advanced algorithm designed to predict impending hypotensive events using arterial waveform analysis and machine learning techniques [3, 4]. Multiple clinical trials have shown that the use of hemodynamic algorithms based on HPI reduced the incidence, duration, and severity of IOH [5, 6]. However, to date, there are no outcome studies directly connecting HPI usage with reduced postoperative complications. For that reason, the Spanish Society of Anesthesiology and Resuscitation (SEDAR), after conducting a structured Delphi questionnaire in 2022 among 30 experts in fluid therapy and hemodynamic monitoring, identified as a research priority comparing intraoperative hemodynamic optimization algorithms based on HPI versus intraoperative hemodynamic management [7]. This study is designed to address this research need.

## Objectives {7}

The primary objective of this multicenter clinical trial is to determine whether hemodynamic management guided by the HPI can reduce the incidence of acute kidney injury (AKI) within 7 days after major elective abdominal surgery.

## Trial design {8}

Multicenter, international, open-label, two-arm, parallel-group randomized controlled trial.

## Methods: participants, interventions, and outcomes

### Study setting {9}

Eligible recruiting sites must have a proven history of performing major elective gastrointestinal surgeries in adult patients. Additionally, they should have the capability to implement hemodynamic therapy with HPI monitoring and a demonstrated track record in participating in interventional research. Although this study is a national initiative from the SEDAR, centers from different countries are also invited to participate. The complete list of participating centers can be found at ClinicalTrials.org.

### Eligibility criteria {10}

This study will include patients over 65 years of age and/or physical condition ASA III or IV. All participants must be scheduled for major elective abdominal surgery (general, urologic, or gynecological surgery), using either a laparoscopic or open surgical approach. A surgery is deemed major if it meets at least one of the following criteria: an expected duration > 2 h or an anticipated blood loss > 15% of the patient's total blood volume.

Patients will be excluded from the study if they have stage 4 or 5 chronic kidney disease (eGFR < 15 mL/min); received a kidney transplant in the past 12 months; are diagnosed with glomerulonephritis, interstitial nephritis, or vasculitis; have anuria at the time of inclusion; have pre-existing AKI; underwent recent renal replacement therapy (RRT) within the past 90 days; require renal replacement at the time of inclusion; are participating in another trial investigating a drug or intervention affecting kidney function; have atrial fibrillation or known cardiac shunts; are undergoing urgent surgery; are pregnant or lactating; are expected to die within 30 days; experienced acute myocardial ischemia or acute pulmonary edema in the previous 30 days; or have any contraindication to low dose of vasoactive or inotropic medication.

Only eligible patients who meet all the inclusion criteria and none of the exclusion criteria and provide voluntary written informed consent will be included in the study.

### Who will take informed consent? {26a}

Before trial enrollment, each participant will provide written informed consent. This procedure includes giving an information sheet to the patient, an associated consent form, and a thorough explanation of the trial’s objectives, methods, potential benefits, and risks. Patients unable to give or withhold informed consent will be not included in the trial. For eligible patients who do not participate in the trial, a detailed record including reasons for their non-participation will be kept.

### Additional consent provisions for collection and use of participant data and biological specimens {26b}

No additional consent provisions are required for the collection and use of data from participants and biological specimens in ancillary studies.

### Interventions

#### Explanation for the choice of comparators {6b}

In this study, the chosen comparator is standard intraoperative blood pressure management, grounded in conventional monitoring and clinical judgment. This choice is well-founded considering the prevailing clinical practices and the need to evaluate the potential benefits of HPI-guided hemodynamic management. The use of standard clinical judgment and conventional monitoring devices is common practice in the participating centers for managing intraoperative blood pressure during elective major abdominal surgeries. This practice involves intermittent or invasive or not invasive continuous blood pressure measurements and clinical assessment by anesthesiologists or healthcare providers. These professionals make real-time decisions on fluid and vasoactive administration based on their evaluations.

By selecting this conventional approach as the comparator, we aim to determine if HPI-based hemodynamic management, which involves a protocolized approach to administering intravenous fluids, inotropic, and/or vasoactive drugs, leads to improved clinical outcome. Specifically, this study aims to demonstrate if HPI guidance significantly reduces the incidence of moderate to severe AKI within 30 days post-surgery when compared to standard clinical judgment and conventional monitoring.

Noteworthy, the control group receiving standard care allows clinicians to exercise their judgment and manage hemodynamics as they deem fit. This might introduce variations across different centers. The study’s comparator choice aims to robustly assess the HPI-based intervention while reflecting the current clinical context and variations in practice across participating centers. By comparing the HPI-based approach with this conventional method, we seek to determine if HPI guidance offers a valuable improvement in clinical practice.

#### Intervention description {11a}

The trial intervention period will begin at the start of surgery, with the skin incision, and will end with the completion of surgery and closure of the skin.

Patient care protocols have been deliberately kept broad in their definition to prevent either overly conservative or misaligned clinical practices.

##### Control group

Patients in this group will undergo treatment as per standard clinical practice. The administration of fluids, vasoconstrictors, and/or inotropic drugs will be at the discretion of the treating clinician. These decisions may be guided by parameters such as heart rate, arterial pressure, diuresis, serum lactate levels, and base excess. While clinicians in the control group can opt to follow a GDHT algorithm by monitoring cardiac output or other hemodynamic variables, the HPI parameter and other hemodynamic variables available from the AcumenIQ sensor will not be integrated into their strategy. In the event of using this technology during the trial intervention period in any patients from the control group, this will be recorded as a protocol violation.

##### Intervention group

The intervention will begin with the induction of general anesthesia and continue until surgery completion. Hemodynamic management will be based on the HPI and advanced functional hemodynamic variables provided by the Hemosphere platform and the AcumenIQ sensor. Non-invasive cuff (AcumenIQ for ClearSight, Edwards Lifesciences, Irvine, USA) or the invasive arterial pressure AcumenIQ sensor (Edwards Lifesciences) could be selected based on individual patient needs. Additionally, both systems offer additional hemodynamic variables such as stroke volume variation (SVV), arterial dP/dtmax, and dynamic arterial elastance (Eadyn), to help identify the most common causes of hypotension.

**Fig. 1:**
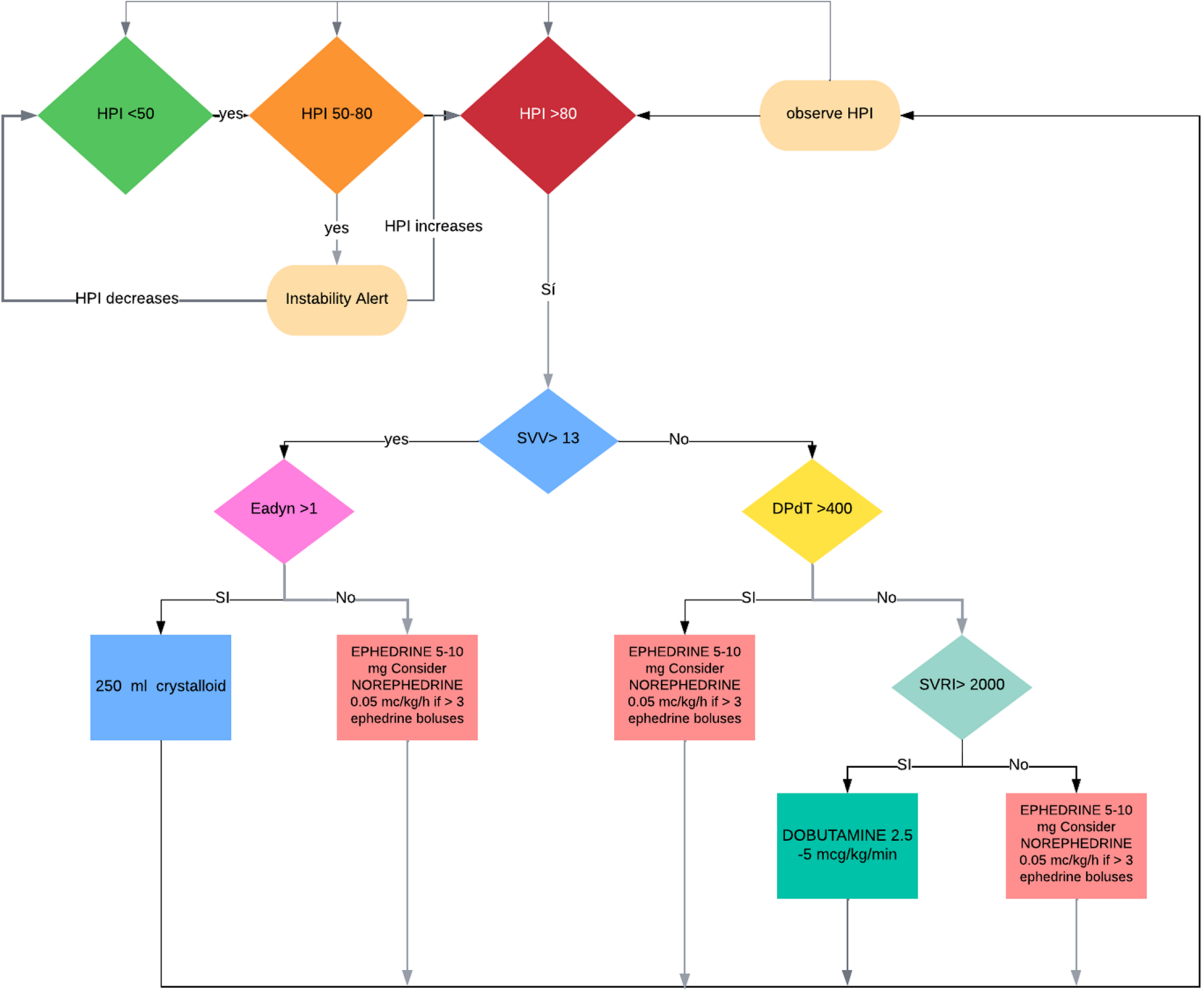
Hemodynamic algorithm

Before initiating continuous blood pressure monitoring and assessing other hemodynamic variables, a maximum of 500 mL of intravenous fluid will be administered. If central venous pressure (CVP) is unavailable, a default value of 5 mmHg will be used to calculate systemic vascular resistance (SVR). The hemodynamic protocol is designed to alert for intervention when the HPI value surpasses 80, although lower values are also considered as a progressive instability warning before reaching that threshold. Once the HPI exceeds 80, therapeutic intervention is advised, involving the administration of fluids and/or vasopressors/inotropes, and is then recommended based on the values of SVV, Eadyn, dP/dtmax, and SVR, as illustrated in Fig. 1.

When the hemodynamic algorithm recommends fluid administration, patients will receive a 250-mL fluid bolus of a balanced crystalloid solution. Ephedrine, administered in bolus doses of 5–10 mg, will be the vasopressor of choice. If continuous infusion is necessary, norepinephrine will be used, always in alignment with the hemodynamic algorithm. Data collection and follow-up for such participants will be performed as usual. All other medical decisions will be the responsibility of the attending physician.

Both study groups will undergo general or combined anesthesia, involving intravenous anesthetic induction and neuromuscular blockade. The choice of anesthesia type will be at the discretion of the attending anesthesiologist for pragmatic reasons.

The selection of the neuraxial analgesia technique, either epidural or intradural, will be based on the preference of the anesthesiologist prior to induction. Intraoperatively, fundamental measures will be implemented to maintain oxygen saturation above 94%, normothermia (body temperature > 36 °C), and a heart rate below 100 beats per minute. Mechanical ventilation will maintain an inspired FiO2 of 60%, targeting a PaCO2 range of 35–45 mmHg, with a tidal volume of 8 ml/kg of ideal body weight and a positive end-expiratory pressure (PEEP) of 4–6 mmHg. Basic monitoring will involve three-lead electrocardiography, pulse oximetry, and at least one peripheral intravenous catheter. Bispectral index (BIS) monitoring, targeting 40–60, will be maintained with sevoflurane or propofol. A maintenance-balanced crystalloid solution will be administered at 1–3 ml/kg/h for laparoscopic procedures and 5–7 ml/kg/h for open surgeries.

Postoperatively, balanced fluid therapy is recommended to meet ion and glucose requirements, with quantities ranging from 1.75 to 2.75 L every 24 h, determined by the responsible clinician. The decision to initiate oral tolerance will also be at the attending clinician’s discretion.

#### Criteria for discontinuing or modifying allocated interventions {11b}

For participants in the intervention group undergoing hemodynamic management guided by the HPI, the potential side effects or adverse reactions from pharmacological components of the hemodynamic protocol warrant careful monitoring. Specific criteria based on safety considerations have been established to modify or discontinue the intervention:

- Tachycardia: Should a participant develop significant tachycardia directly linked to the administration of catecholamines or inotropes as per intervention’s hemodynamic management, their well-being and comfort must be prioritized. In such instances, the intervention might require modification of discontinuation to effectively address the tachycardic response.
- Allergic Reactions: If a participant manifests allergic reactions to any of the pharmacological components of the intervention, this must be halted immediately. Addressing the allergic reactions while ensuring participant safety is paramount. Such occurrences should be documented as adverse events in the trial record.

#### Strategies to improve adherence to interventions {11c}

Following an initial meeting between the principal investigators and all local lead investigators, training sessions will be conducted at the partner hospitals. These sessions will cover the process of randomization, enrollment, data acquisition, and treatment strategies. Recruitment targets will be closely monitored, and regular feedback will be provided to the participating sites to ensure active management and adherence throughout the trial.

#### Relevant concomitant care permitted or prohibited during the trial {11d}

During the HYT study, patients are not allowed to participate in another clinical trial involving modifications to perioperative patient management.

#### Provisions for post-trial care {30}

Patients participating in this study benefit from indemnity against negligent harm through the standard Spanish National Health Service Indemnity arrangements. It’s pertinent to note that this trial is classified as a low-intervention level clinical trial. Within the hemodynamic management protocol, medications are dispensed strictly according to their product data sheet and consistent with the standard clinical practice. The patient management within this study does not introduce any novel risks, and any potential risk aligns with those inherent in routine clinical care.

Healthcare professionals responsible for conducting this trial are either covered by individual or collective professional civil liability insurance or have an equivalent financial guarantee provided from the healthcare institution where the trial will be conducted. For clinical trials classified as low-intervention level clinical trials, there is no obligation for additional insurance coverage. This is because any potential damage arising from the clinical trial is already covered by the civil liability insurance—whether individual or collective—of the healthcare center or institution supervising the clinical trial. The trial sponsor will provide a certificate from the institution’s representative, confirming that the insurance policy of the healthcare center or organization adequately covers clinical trials of this nature. For further information, please refer to the following resource: https://www.aemps.gob (in Spanish).

### Outcomes {12}

#### Primary endpoint

The primary outcome is the occurrence of moderate or severe AKI (according to KDIGO Stage 2–3 criteria [8]) within 7 days after surgery (Table 2).

#### Secondary endpoints

Secondary outcomes:

1. RRT requirement and duration: Occurrence within 30 days post-surgery.
2. Postoperative Complications defined according to the European Perioperative Clinical Outcome (EPCO) [9] definitions within 30 days post-surgery.
3. Reoperation within 30 days post-surgery.
4. Unplanned Intensive Care Admission for the treatment of one or more complications within 30 days of randomization.
5. 30-day mortality.

Planned process measures:

1. Duration of hospital stay (number of days from randomization until hospital discharge).
2. Duration of critical care stay (number of days from randomization until hospital discharge).

### Participant timeline {13}

Participants will be enrolled in the study during the pre-study period, which includes both the screening and recruitment of eligible individuals. Once enrolled, a baseline assessment will be conducted, during which informed consent will be obtained, and baseline data will be collected.

On day 0, participants will undergo randomization. The study intervention will start with the onset of the surgery (skin incision) and conclude upon its completion (skin closure). From day 0 through the duration of their hospital stay, participants will be evaluated for the primary and secondary outcomes.

On day 30, participants will return for a follow-up evaluation, where 30-day outcomes will be assessed, and the study will be completed (Table 1).

**Table 1:** Participant timeline

**Event/Visit**	**Revision**	**Preoperative**	**Surgery**	**Hospital discharge**	**30 days after IQ**
Criteria of inclusion/exclusion	x				
Informed consent	x				
Demographic information		x			
Medical history		x			
Height and weight		x			
Randomization		x			
Intraoperative			x		
Hemodynamic algorithm			x		
Review of medical notes			x	x	x
ICU days and hospitalization				x	
Follow-up					x
Notification of adverse effects			x	x	x

### Sample size {14}

To detect an absolute reduction of 5% in the incidence of AKI within 30 days after surgery, from an assumed baseline rate of 10% to a target rate of 5%, with a 1:1 allocation ratio, and maintaining an overall type I error rate of 5%, with an anticipated dropout rate of 10%, a total of 958 patients (479 per arm) are required to achieve an 80% statistical power [10].

### Recruitment {15}

To ensure adequate participant enrollment, our strategies include ensuring the attainment of the target number of appropriate recruitment sites, maintaining coordinated national leadership, actively engaging local anesthesiologists to support the screening and execution of the trial, and carefully selecting sites with highly experienced local investigators proficient in HPI utilization and well-equipped research teams.

## Assignment of interventions: allocation

### Sequence generation {16a}

Potential participants will be screened by research staff from pre-admission clinic lists and operating theater schedules. Eligible participants will be identified and approached by a research team member before the scheduled surgery, preferably at least 24 h in advance. After obtaining written informed consent, patients will be randomized before the scheduled surgical procedure.

In order to ensure the homogeneity of the patient groups included in the treatments, a pseudo-random process will be carried out for the assignment of patients to these treatment groups. This process will be conducted in blocks of 4, distributed across segments defined by center, age group (under 75 years and 75 years or older), and patient's risk level (High risk, low risk).

This randomization will be managed centrally via an online platform (REDCap, Research Electronic Data Capture) [11]. Treatment group assignment will remain confidential until the randomization process is completed.

### Concealment mechanism {16b}

Participants will be randomized using the REDCap platform. Allocation concealment is ensured, as this platform will not release the randomization code until all prerequisites are met, including checking the inclusion and exclusion criteria, obtaining the patient's informed consent, and completing all baseline measurements.

### Implementation {16c}

Consenting patients meeting the inclusion criteria and none of the exclusion criteria will be randomized. The principal investigator at each center will be responsible for requesting randomization. This method is unpredictable, thereby minimizing the risk of selection bias among research staff enrolling participants.

## Assignment of interventions: blinding

### Who will be blinded {17a}

This trial will be conducted in an open-label manner, so both participants and intervention-administering staff will be aware of the treatment group assignments. Nonetheless, measures will be implemented to minimize potential bias arising from the research staff's knowledge of treatment allocation. To ensure unbiased clinical outcome assessments, the principal investigator at each center will be responsible for these evaluations and will remain detached from participant care and blinded to treatment group assignments. Similarly, individuals interacting with participants during follow-up will also be unaware of treatment group allocations.

The trial steering committee will not have access to results broken down by the treatment group throughout the trial. The final analysis will take place after all follow-up data has been collected, the final statistical analysis plan has been approved, and data cleaning is complete. The independent data monitoring committee will have access to outcome results by treatment group, but data will be handled by an independent statistician not otherwise involved in the trial.

### Procedure for unblinding if needed {17b}

Unblinding will only be considered when any treating physician or regulatory authority believes that it is essential to know the treatment group to which the patient belongs. In general, unblinding of participants during clinical trial development is not permitted, unless there are compelling medical or safety reasons to do so.

Emergency unblinding will be performed by identifying the patients belonging to the experimental or control groups by reviewing the list containing the group to which each patient has been randomized, supervised by the principal investigator of each center.

Investigators should diligently uphold blinding, disclosing the actual allocation to neither the patients nor any other study personnel, including site staff, monitors, or the steering committee. There should be no written or verbal revelation of the allocation code in any patient-related documentation. Unblinding will only be considered when any treating physician or regulatory authority believes that it is essential to know the treatment group to which the patient belongs. In general, unblinding of participants during clinical trial development is not permitted, unless there are compelling medical or safety reasons to do so.

Emergency unblinding will be performed by identifying the patients belonging to the experimental or control groups by reviewing the list containing the group to which each patient has been randomized, supervised by the principal investigator of each center.

## Data collection and management

### Plans for assessment and collection of outcomes {18a}

The perioperative variables to be collected will be listed in Table 2.

**Table 2:** Study variables

Age
Sex
Height
Weight
ASA physical score
Diagnosis of chronic lung disease (COPD, asthma, interstitial lung disease)
Diagnosis of ischemic heart disease
Diagnosis of diabetes mellitus
Diagnosis of heart failure
Diagnosis of liver cirrhosis
Diagnosis of active cancer (indication for surgery Y/N)
Diagnosis of previous stroke or transient ischemic attack
Current smoker (has smoked in the last 14 days)
Preoperative hemoglobin
Intraoperative Variables
Total fluid therapy during surgery: types and total amounts (ml)
Cumulative dose during the intraoperative period of vasoactive agents: specify by drugs used (mg)
Accumulated dose during the intraoperative period of ionotropic drug in case of indication (mg)
Total intraoperative diuresis (ml)
Estimated bleeding (ml)
Transfusion of total blood products during surgery
Use of cardiac output monitor
Using beat-to-beat blood pressure measurement
Related to the Intervention
Hospital admission date
Surgery date
Type of anesthesia with which the intervention is performed
Surgical technique
Anesthesia duration
Duration of surgical intervention
AKI > I at 7 days
Postoperative variables, complications, and mortality
Presence of postoperative treatment with: diuretics, ACE inhibitors, angiotensin II receptor blocker (ARB), beta-blockers, NSAIDs, and other postoperative nephrotoxic drugs, as well as daily fluid therapy
Length of hospital stay
30-day mortality
Tracking Data
24-h and 30-day adverse cardiac events (≥ Clavien-Dindo grade II)
Other 30-day postoperative complications
Red blood cell transfusion within 30 days of surgery
Endoscopic or radiological intervention within 30 days of randomization
Reoperation within 30 days of randomization
Unplanned intensive care admission to treat one or more complications within 30 days of randomization
Prolonged scheduled intensive care admission due to one or more complications within 30 days of randomization
Invasive mechanical ventilation after leaving the operating room, within 30 days of randomization
Date of death (if applicable)

The KDIGO (Kidney Disease: Improving Global Outcomes) is an organization that provides guidelines and recommendations to enhance the care of patients with kidney disease. Within the context of AKI definitions, the KDIGO has established specific criteria for identifying and classifying AKI [8].

To assess the presence of postoperative AKI, we recommend a standardized protocol for evaluating renal function across all participating centers. This protocol comprises two key components: the measurement of serum creatinine and the assessment of diuresis.

Prior to surgery, a blood sample is collected from the patient to measure serum creatinine at a preoperative time point corresponding to the preoperative visit. These samples are sent to the clinical laboratory of each center for analysis. Subsequently, post-surgery, serum creatinine measurements are taken at various time points, including the immediate postoperative period, followed by regular 24-h intervals while the patient remains at the center during the first 7 days after surgery. Mandatory measurements for the study are taken on the day of the surgery and the first postoperative day, with recorded serum creatinine values. The evaluation of diuresis is conducted post-surgery, commencing data collection immediately after the procedure. The amount of urine produced is measured and recorded at regular intervals, typically every hour, for the initial 24 h. Urinary flow rate is calculated in milliliters per hour (mL/h), and diuresis values times are documented.

The protocol defines clear criteria for interpreting results, with particular attention to significant increases in serum creatinine and low urinary flow rates. The definition of postoperative complications is provided in Table 3.

**Table 3:** Postoperative complications at 30 days after surgery [9]

**Complication**	**Definition**	**Severity scale**
**Acute kidney injury (on 8 to 30 days)**	- *Mild: *Serum creatinine elevation 1.5–1.9 times above baseline in 7 days or ≥ 0.3 mg/dL (30 μmol/L) in 48 h. Diuresis ≤ 0.5 ml/kg/h for 6–12 h - *Moderate: *Elevation of serum creatinine 2.0–2. 9 times above baseline in 7 days. Diuresis ≤ 0.5 ml/kg/h for 12 h - *severe:*Elevation of serum creatinine 3 times above baseline in 7 days or increase in serum creatinine ≥ 4.0 mg/dL (≥ 350 μmol/L) with an acute elevation of > 0.5 mg/dL (> 50 μmol/L) or initiation of renal replacement therapy. Diuresis ≤ 0.3 ml/kg/h for 24 h or anuria for 12 h	Included in the definition
**Acute respiratory distress syndrome (ARDS)**	Respiratory failure, or new or worsening respiratory symptoms, beginning within the first week after surgery; and a chest x-ray or CT scan demonstrating bilateral opacities not fully explained by effusions, lobar/pulmonary collapse, or nodules; and respiratory failure is not fully explained by heart failure or fluid overload Needs objective evaluation (e.g., echocardiography) to exclude hydrostatic edema if no risk factor is present	- *Mild:* PaO2:FiO2 between 200 and 300 mmHg with PEEP or CPAP ≥ 5 cmH2O - *Moderate*: PaO2:FiO2 between 100 and 200 mmHg and PEEP ≥ 5 cmH2O - *Serious*: PaO2:FiO2 ≤ 100 mmHg with PEEP ≥ 5 cmH2O
**Pneumonia**	Chest radiographs showing new or progressive and persistent infiltrates, or consolidation, or cavitation, and at least one of the following: a) Fever (> 38 °C) with no other known cause b) Leukopenia (< 4000 leukocytes/mm^3^) or leukocytosis (> 12,000 leukocytes/mm^3^) c) In adults > 70 years, altered mental status without any other recognized cause …and at least two of the following: - New appearance of purulent sputum or change in sputum characteristics, or increased respiratory secretions, or increased suction demands - New onset or worsening cough, or dyspnea, or tachypnea - Râles or bronchial breath sounds - Worsened gas exchange (hypoxia, increased oxygen, or ventilator demand)	- *Mild:* it produces only temporary damage and would generally not require specific clinical treatment - *Moderate:* more serious complication, but one that does not usually cause permanent damage or functional limitation. Usually requires clinical treatment - *Serious:* produces a significant prolongation of hospital stay and/or permanent functional limitation or death. It almost always requires clinical treatment
**Cardiac arrest**	Cessation of cardiac mechanical activity, as confirmed by the absence of signs of circulation. ECG changes may confirm cardiac arrest	None: Binary (yes/no)
**Arrhythmia**	Electrocardiographic (ECG) evidence of abnormal heart rhythm	- *Mild:* it produces only temporary damage and would generally not require specific clinical treatment - *Moderate:* more serious complication, but one that does not usually cause permanent damage or functional limitation. Usually requires clinical treatment - *Serious:* produces a significant prolongation of hospital stay and/or permanent functional limitation or death. It almost always requires clinical treatment
**Deep venous thrombosis**	A new blood clot or thrombus within the venous system. A systematic review is required in trials in which DVT is an important outcome measure. Appropriate diagnostic tests include ultrasonography, venography, computed tomography, or magnetic resonance imaging	
**Stroke**	Embolic, thrombotic, or hemorrhagic cerebral event with persistent residual motor, sensory, or cognitive dysfunction (e.g., hemiplegia, hemiparesis, aphasia, sensory deficit, impaired memory)	
**Pulmonary edema**	Evidence of fluid accumulation in the alveoli due to impaired cardiac function	- *Mild:* it produces only temporary damage and would generally not require specific clinical treatment - *Moderate:* more serious complication, but one that does not usually cause permanent damage or functional limitation. Usually requires clinical treatment - *Serious:* produces a significant prolongation of hospital stay and/or permanent functional limitation or death. It almost always requires clinical treatment
**Pulmonary embolism**	A new blood clot or thrombus within the pulmonary arterial system Guidance: Appropriate diagnostic tests include scintigraphy and computed tomography angiography. Measurement of plasma D-dimer is not recommended as a diagnostic test in the first three weeks after surgery	
**Surgical site infection (superficial)**	Infection involving only the superficial surgical incision, meeting the following criteria: 1) Infection occurs within 30 days after surgery and 2) Involves only the skin and subcutaneous tissues of the incision and 3) The patient has at least one of the following: a) Purulent drainage from the superficial incision b) Organisms isolated from a fluid or tissue culture obtained aseptically from the superficial incision and at least one of the following signs or symptoms of infection: pain or tenderness, localized swelling, redness or warmth, or superficial incision deliberately opened by the surgeon and is culture positive or not cultured. A negative culture does not meet this criterion c) Diagnosis of an incisional surgical site infection by a surgeon or GP	
**Surgical site infection (deep)**	An infection involving superficial and deep parts of the surgical incision and meeting the following criteria: 1) Infection occurs within 30 days after surgery if a surgical implant is not left or 1 year if the implant is in place and 2) The infection appears to be related to the surgical procedure and involves the soft tissues deep to the incision (for example, the fascial and muscular layers) and 3) The patient has at least one of the following: a) Purulent drainage from the deep incision but not from the organ/space component of the surgical site b) The surgeon opens a deep incision spontaneously or is deliberately opened and has a positive culture or no cultures were performed while the patient has at least one of the following signs or symptoms of infection: fever (> 38 °C) or localized pain or sensitivity. A negative cultural finding does not meet this criterion c) An abscess or other evidence of infection involving the deep incision is found on direct examination, during surgery, or on histopathological or radiological examination d) Diagnosis of a deep incisional infection at the surgical site by a surgeon or treating physician	- *Mild:* it produces only temporary damage and would generally not require specific clinical treatment - *Moderate:* more serious complication, but one that does not usually cause permanent damage or functional limitation. Usually requires clinical treatment - *Serious:* produces a significant prolongation of hospital stay and/or permanent functional limitation or death. It almost always requires clinical treatment
**Surgical site infection (organ/space)**	An infection that involves any part of the body excluding the fascia or muscle layers and meets the following criteria: 1) Infection occurs within 30 days after surgery and 2) The infection appears to be related to the surgical procedure and involves any part of the body, excluding the incision in the skin, fascia, or muscle layers, that is opened or manipulated during the surgical procedure, and 3) The patient has at least one of the following: a) Purulent drainage from a drain that is placed through an incision in the organ/space b) Organisms isolated from aseptically obtained fluid or tissue culture in the organ/space c) An abscess or other evidence of infection involving the organ/space found on direct examination, during reoperation, or on histopathological or radiological examination d) Diagnosis of an organ/space surgical site infection by a surgeon or treating physician	
**Bacteremia**	An infection that is not related to an infection at another site and that meets any of the following criteria: 1) The patient has a recognized pathogen cultured from blood cultures that is not related to an infection at another site 2) The patient has at least one of the following signs or symptoms: fever (> 38 °C), chills, or hypotension and at least one of the following: a) Common skin contaminant cultured from two or more blood cultures drawn on separate occasions b) Common skin contaminant that is cultured from at least one blood culture from a patient with an intravascular line, and antimicrobial therapy is started by a physician c) Positive blood antigen test	- *Mild:* Causes only temporary damage and generally does not Mild: Causes only temporary damage and would generally not require specific clinical treatment - *Moderate:* more serious complication, but one that does not usually cause permanent damage or functional limitation. Usually requires clinical treatment - *Serious:* produces a significant prolongation of hospital stay and/or permanent functional limitation or death. It almost always requires clinical treatment
**Myocardial infarction**	Increased plasma cardiac biomarker values (preferably cardiac troponin) with at least one value above the 99th percentile upper reference limit and at least one of the following criteria: - Symptoms of ischemia - New or suspected new ST-segment or T-wave ECG changes or new left bundle branch block - Development of pathological Q waves on ECG - Radiological or echocardiographic evidence of new loss of viable myocardium or new regional wall motion abnormality - Identification of an intra-coronary thrombus on angiography or autopsy	
**Urinary tract infection**	An infection associated with at least one of the following signs or symptoms that must be identified within a 24-h period: fever (> 38 °C), urgency, frequency, dysuria, suprapubic tenderness, costovertebral angle pain, or tenderness without other cause recognized and a positive urine culture of ≥ 105 colony-forming units/mL with no more than two species of microorganisms	
**Paralytic ileus**	Not tolerate solid food or bowel movements for three or more days after surgery	
**Delirium**	Delirium can be identified using the Intensive Care Delirium Screening Checklist Patients are first evaluated for an altered level of consciousness. Those with a mild or moderate response to stimulation, an exaggerated response to stimulation, or normal wakefulness are fully evaluated. Patients receive one point for each of the following criteria: inattention, disorientation, hallucinations, psychosis, agitation or psychomotor retardation, inappropriate language or mood, sleep/wake cycle disturbance, or fluctuating symptoms	Built into the definition
**Postoperative hemorrhage**	Blood loss that occurs within 72 h of the end of surgery, which would normally result in a blood transfusion	- *Mild:* any sign of bleeding (any bleeding that is more than expected, including bleeding only identified on imaging), that does not meet the criteria for moderate-severe type, but requires at least one of the following: • Non-surgical medical intervention by a healthcare professional (examples include stopping antiplatelet, antithrombotic medications, compression at bleeding site, use of reversal medications such as: protamine and vitamin k) • Requires hospitalization or higher level of care • It requires rapid evaluation with tests such as: complete blood count, urinalysis, coagulation tests, endoscopy and tomography - *Moderate:* • Bleeding with a decrease in hemoglobin of ≥ 3 to < 5 g/dl (related to bleeding) • Any need for transfusion due to obvious bleeding • Decrease in hemoglobin ≥ 5 g/dl (related to bleeding) • Bleeding that requires surgical intervention for its control • Bleeding requiring the use of vasoactive agents - *Severe:* Transfusion of ≥ 5 units of red blood cells, in a period of 48 h. fatal bleeding

### Plans to promote participant retention and complete follow-up {18b}

N/a: This is not required, as patient follow-up is conducted through the review of medical records.

### Data management {19}

Data entry is seamlessly managed through the REDCap system. This can be initiated at each participating site, where the data originates, or at a dedicated Core Coordinating Center (Hospital Universitario Infanta Leonor, Madrid, Spain). The initial study forms are entered and retained at their respective originating sites.

#### Data transmission and editing

REDCap’s electronic data entry screens precisely replicate the approved paper forms sanctioned by the Steering Committee. Data integrity is meticulously upheld through several mechanisms, encompassing referential data rules, valid values, range checks, and consistency checks against pre-existing database data (longitudinal checks). Users are given the option to select values from a list of valid codes, supported by descriptions for each code where applicable. Data checks are rigorously applied during data entry into specific fields and before data is permanently committed to the database. Any modifications to the stored data are comprehensively documented through either the data change system or an inquiry system. The activities of users are regulated by the privileges linked to their unique user identification codes and passwords.

#### Data discrepancy inquiries and reports

Specially designed programs are employed to pinpoint errors such as missing data or specific inconsistencies. These errors are succinctly summarized in Data Query Reports, which are then dispatched to Data Managers at the Core Coordinating Centers. When Data Managers receive inquiries, they meticulously cross-check the original forms to address inconsistencies and consult alternative sources for any necessary corrections. Simultaneously, the original paper forms are adapted accordingly, and responses to the inquiries are included.

#### Security and data back-up

To ensure the security of data, all study-related forms, diskettes, and tapes are securely stored within locked cabinets. Stringent access controls are in place, managed by a password system with regular password updates. Reports are meticulously prepared to protect the confidentiality of individual subjects. Data is systematically backed up, including a comprehensive backup of the primary database performed bi-monthly, and securely retained off-site indefinitely. Daily incremental data backups are maintained on-site, and periodic data analysis files are also backed up. Additional measures for regular data backup and export are systematically carried out at the database management level.

#### Study status reports

The Data Coordinating Center consistently issues weekly email reports containing details regarding missing data, incomplete forms, and absent visits. Personnel at both the Core Coordinating Center and Participating Sites diligently review these reports to ensure accuracy and promptly report any discrepancies to the Data Coordinating Center.

### Confidentiality {27}

All patients included in the study, that is, those who have signed the informed consent for the study, after being randomized, will be identified with a sequential alphanumeric identification code that will be assigned to each patient according to the correlative order of inclusion. The sequential numerical code will be generated by the electronic application used to perform the randomization. The list with the identification data of each patient associated with the identification code of the database will only be accessible by local researchers and will be guarded by the principal investigator of each center.

Personal data will be treated confidentially, as established by Spanish Organic Law 3/2018, of December 5, on the Protection of Personal Data and guarantee of digital rights. In the case of transmission of the personal data of Spanish patients to a third State, the European regulations on the protection of personal data will be complied with.

### Plans for collection, laboratory evaluation, and storage of biological specimens for genetic or molecular analysis in this trial/future use {33}

N/a: In this trial, the collection, laboratory evaluation, and storage of biological specimens for genetic or molecular analysis in the future are not envisaged.

## Statistical methods

### Statistical methods for primary and secondary outcomes {20a}

The analyses will follow the intention-to-treat principle, including all participants with recorded outcomes, and will be based on their randomized treatment allocation.

Categorical variables will be presented with absolute values and relative frequencies, while continuous variables will be described using means and standard deviations for normally distributed data and medians with interquartile ranges (IQR) (25th to 75th percentiles) for non-normally distributed data. The Kolmogorov–Smirnov test will be used to assess the distribution of the data. Treatment effects, 95% confidence intervals (CIs), and *p*-values will be provided for both primary and secondary outcomes, as well as for process measures according to each treatment group. However, baseline and follow-up data for both groups will be summarized based on the treatment received, and statistical testing will not be applied.

The analysis for the primary outcome will follow the intention-to-treat principle, in which all the randomized patients will be analyzed in the assigned group. The efficacy of the intervention regarding the primary outcome of AKI within 7 days will be assessed using a mixed-effects logistic regression model. In this model, institutions will be treated as random intercepts to account for variations in clinical practice across different participating centers. Detailed reports, including relative risks (RR), their respective 95% confidence intervals (CIs), *p*-values, absolute frequencies, and relative percentages, will be provided for both the intervention and control groups. The model will also adjust for surgical procedure category, age, sex, ASA grade, and initial creatinine level. ASA grade and surgical procedure category will be introduced as categorical variables.

Regarding secondary outcomes, univariate analyses will compare categorical variables between the intervention and control groups. Mixed-effects regression models will be employed for each postoperative complication, treating institutions as random intercepts. The models will account for additional variables, including surgical procedure, age, sex, ASA grade, and baseline creatinine. Continuous variables will be analyzed using time-to-event models with competing risks due to mortality upon discharge from the hospital. The findings will be presented as mean differences along with their corresponding 95% CIs and *p*-values.

All statistical analyses will be conducted using Stata version 14.0 (Stata College, TX, USA).

### Interim analyses {21b}

No interim analyses were conducted due to resource optimization, ethical considerations, and the robust design of the study. With clear and stable endpoints, and sufficient statistical power, we prioritize a comprehensive analysis at the study’s conclusion to streamline processes and ensure efficient resource utilization.

### Methods for additional analyses (e.g., subgroup analyses) {20b}

Subgroup analyses will be performed based on surgical modality, patient risk stratification (ASA ≥ III versus ASA I/II), age (elderly > 65 years old vs younger), and other preoperative comorbidities (diabetes, chronic lung disease, hypertension). We will also perform subgroup analysis based on invasive vs non-invasive hemodynamic monitoring (presence of arterial line). Statistical tests will be adjusted for multiple comparisons using Bonferroni correction.

### Methods in analysis to handle protocol non-adherence and any statistical methods to handle missing data {20c}

Missing baseline data will be addressed through mean imputation. Sensitivity analyses will be performed in cases where over 1% of AKI data is missing; sensitivity analyses will be conducted using imputation models. These models will assume varying AKI incidences depending on the baseline incidence in each group (μ): *μ* − − 4%, *μ* − − 2%, *μ* + 2%, *μ* + 4%. For each combination, the imputation model will randomly assign patients with missing AKI data.

The patients from the centers that do not recruit a minimum of 10 patients will be excluded from the study, with the possibility of exceptions to be assessed by the Steering Committee.

This requirement is set to ensure adequate sample size and statistical power. When centers recruit fewer than 10 patients, it can significantly impact the study’s ability to draw reliable conclusions.

### Plans to give access to the full protocol, participant-level data, and statistical code {31c}

The data that support the findings of this study are available from the corresponding author [JRM] upon reasonable request.

## Oversight and monitoring

### Composition of the coordinating center and trial steering committee {5d}

Principal Investigator and Research Physician

- Design and conduct of HYT
- Preparation of protocol and revisions
- Preparation of investigators brochure (IB) and CRFs [Case Report Forms]
- Organizing steering committee meetings
- Publication of study reports

Steering committee (SC)

(see title page for members)

- Agreement of final protocol
- Reviewing the progress of the study and if necessary agreeing to changes to the protocol and/or investigators brochure to facilitate the smooth running of the study.

Trial Management Committee (TMC)

- Study planning
- Organization of steering committee meetings
- Responsible for trial master file
- Budget administration and contractual issues with individual centers
- Advice for lead investigators
- Assistance with ethics committee applications
- Data verification

Data Manager

- Maintenance of trial IT system and data entry
- Data verification

### Lead Investigators

In each participating center, a lead investigator will be identified, to be responsible for identification, recruitment, data collection, and completion of CRFs, along with follow-up of study patients and adherence to study protocol and investigators brochure.

### Explanation

#### Composition of the data monitoring committee, its role and reporting structure {21a}

An Independent Data Monitoring Committee (IDMC) has been appointed to oversee the safety and efficacy of the interventions during the recruitment period of the trial. The IDMC will review patient recruitment, data quality, protocol compliance, and loss to follow-up. No formal interim analysis for efficacy has been scheduled.

#### Protocol adherence monitoring and safety monitoring

Predefined protocol deviations that will be reported include instances where Acumen monitoring is either not utilized in an intervention group participant or inappropriately used in the control group. We will closely monitor protocol deviations and provide feedback to centers exhibiting significant non-compliance. All interventions in the HYT trial are already part of routine clinical practice for patients undergoing major abdominal surgery in participating centers. Furthermore, each recruitment site will have initiation visits. Each center will have monitoring visits during the trial recruitment period, and adherence to the hemodynamic protocol will be evaluated. Full source data verification will be conducted.

### Adverse event reporting and harms {22}

The principal investigator bears responsibility for adverse event classification and the ongoing evaluation of clinical research safety. The principal investigator’s duties include:

- Reviewing the investigator’s assessment of all adverse events and documenting their severity and relationship to the investigational product in case of any disagreement between the principal investigator(s) and the sponsor. Both assessments should be communicated to the relevant parties.
- Reviewing all product deficiencies and determining if they could have caused a serious adverse product effect, with the option to communicate differing assessments to the concerned parties.
- Reporting or ensuring that the principal investigator(s) report all serious adverse events and product deficiencies that could have led to a serious adverse product effect.
- Notifying regulatory authorities of all serious adverse events and product deficiencies that could have caused a serious adverse product effect within the required timeframe as mandated by national regulations.
- Evaluating whether the risk analysis necessitates an update and assessing whether corrective or preventive actions are needed for serious adverse product effects and product deficiencies that could have caused serious adverse product effects.

Given that the investigational product is a marketed product used under its marketing conditions, the European medical device surveillance system’s requirements will be considered for serious adverse event notifications to avoid possible duplications.

The Principal Investigator must:

- Record each adverse event and observed product deficiency along with an assessment, and promptly notify the sponsor of all serious adverse events
- Promptly notify the sponsor of all serious adverse events and product deficiencies that could have caused a serious adverse product effect, except for those specified in this clinical investigation plan as events not requiring immediate communication
- Provide the sponsor, upon request, with additional information related to the safety report of a specific adverse event
- Report suspicions of serious and unexpected adverse reactions to AEMPS via Eudravigilance_CTM, rather than to the Ethics Committee, with the narrative of cases being acceptable in either English or Spanish, preferably accompanied by an English summary
- Not submit biannual reports regarding serious and unexpected adverse reactions
- No longer communicate suspected serious and unexpected adverse reactions (SUSAR) or annual safety reports to the health authorities of the Autonomous Communities, effective January 31, 2022

This research employs a medical device with European Community marking, used under its marketing conditions. Consequently, only adverse events directly related to the use of the product under investigation will be considered for reporting. Adverse events occurring during the surgical procedure and the postoperative period will not be considered as such for reporting purposes.

### Frequency and plans for auditing trial conduct {23}

Frequency and Plans for Auditing Trial Conduct:

The Project Management Group, comprising members of the Trial Steering Group and located at Infanta Leonor University Hospital, played a central role in the daily coordination of the study. Although initially ad hoc meetings were conducted as needed, the team later adopted a structured approach, scheduling regular meetings to holistically review trial conduct. These sessions were flexibly organized to align with the study’s evolving needs, ensuring a responsive and adaptive trial management strategy. Simultaneously, the Trial Steering Group and the independent Data Monitoring Committee convened regularly to maintain comprehensive oversight of trial conduct throughout the study period. The study protocol, specifically detailed in point 21a, establishes a Data Monitoring Committee to ensure the ongoing safety and efficacy of interventions.

Patient Public Involvement:

No specific Patient Public Involvement was incorporated into the design or implementation of this study.

Composition and Roles of Coordinating Center, Trial Steering Committee, and Trial Management Committee:

The Project Management Group assumed a key role in day-to-day trial operations, ensuring organizational support. While the meetings were initially organized on an ad hoc basis, they transitioned into regular sessions, fostering effective communication and coordination. Specific details on the Trial Steering Committee, Trial Management Committee, and their roles are outlined in the study protocol (5d).

### Plans for communicating important protocol amendments to relevant parties (e.g., trial participants, ethical committees) {25}

Any modifications to the protocol which may impact the conduct of the study and potential benefit of the patient or may affect patient safety, including changes in study objectives, study design, patient population, sample sizes, study procedures, or significant administrative aspects, will require a formal amendment to the protocol. If it is necessary to make a modification to the protocol, this modification must be agreed between the principal investigator and the sponsor and signed by both parties in a new version of the protocol. Such an amendment will be agreed upon by the SC and approved by the Ethics Committee prior to implementation and notified to the health authorities in accordance with local regulations.

## Dissemination plans {31a}

The study results will be released to the participating physicians, referring physicians, and the general medical community.

## Discussion

Our primary focus revolves around assessing the efficacy of hemodynamic management guided by the HPI, which entails the protocolized administration of intravenous fluids, inotropic, and vasoactive agents, in patients undergoing elective major abdominal surgery. Our aim is to determine whether this approach can effectively reduce the incidence of moderate to severe AKI within 7 days after surgery. To date, the association between intraoperative hypotension, as predicted by the HPI and its impact on postoperative complications, has not been conclusively established in randomized clinical trials [6, 12]. Therefore, the need for a large-scale, multicenter clinical trial becomes apparent. This study is designed to address whether the implementation of an HPI-based hemodynamic protocol can indeed lead to improved postoperative clinical outcomes.

However, it is crucial to acknowledge several limitations in our study design. One of the inherent challenges stems from the fact that our control group will not receive a protocolized treatment. This arrangement provides clinicians in the control group with the autonomy to manage hemodynamics based on their clinical judgment. Consequently, this approach may lead to variations in patient care and management practices across different participating centers, which can introduce confounding variables and potentially influence the outcomes. Furthermore, the lack of a standardized control protocol could potentially affect the interpretation of results and the ability to draw definitive conclusions about the impact of HPI-based guidance. Additionally, the study’s findings will be influenced by the knowledge and experience of the clinical teams responsible for the patient’s care. Variations in clinical practices and decisions may arise due to individual preferences and center-specific approaches. Nevertheless, a noteworthy strength of our study is the transparent and independent nature of our Delphi process among experts [7], contributing robustness and reliability to our findings. This inclusion underscores the impartiality and freedom inherent in our process, enhancing the quality of the results obtained.

## Trial status

HYT trial (Version 1, April 2022) Recruitment started in October 2022 and was completed in January 2023.

## Data Availability

from the corresponding author on reasonable request.

## Abbreviations

AEMPS: Agencia Española del Medicamento y Productos Sanitarios
AKI: Acute kidney injury
ASA: American Society of Anesthesiologists score
BIS: Bispectral index
CI: Confidence interval
CRF: Case Report Forms
CVP: Central venous pressure
DO_2_: Oxygen delivery
Eadyn: Dynamic arterial elastance
EPCO: European Perioperative Clinical Outcome
GDHT: Goal-directed hemodynamic therapy
GFR: Glomerular filtration rate
HPI: Hypotension prediction index
IB: Investigators brochure
IDMC: Independent Data Monitoring Committee
IQR: Interquartile ranges
KDIGO: Kidney Disease Improving Global Outcomes
PEEP: Positive end-expiratory pressure
REDCap: Research Electronic Data Capture
RRT: Renal replacement therapy
SC: Steering committee
SEDAR: Spanish Society of Anesthesiology and Resuscitation
SUSAR: Suspected serious and unexpected adverse reactions
SVR: Systemic vascular resistance
SVV: Stroke volume variation
TMC: Trial Management Committee
